# An Angle of Polarization (AoP) Visualization Method for DoFP Polarization Image Sensors Based on Three Dimensional HSI Color Space

**DOI:** 10.3390/s19071713

**Published:** 2019-04-10

**Authors:** Hui Wang, Haofeng Hu, Xiaobo Li, Zijian Guan, Wanshan Zhu, Junfeng Jiang, Kun Liu, Tiegen Liu

**Affiliations:** 1 School of Precision Instrument & Opto-Electronics Engineering, Tianjin University, Tianjin 300072, China; whoptic@163.com (H.W.); lixiaobo@tju.edu.cn (X.L.); guanzjam@163.com (Z.G.); zhuwanshan@tju.edu.cn (W.Z.); jiangjfjxu@tju.edu.cn (J.J.); beiyangkl@tju.edu.cn (K.L.); tgliu@tju.edu.cn (T.L.); 2 Key Laboratory of Opto-Electronics Information Technology, Ministry of Education, Tianjin 300072, China; 3 Tianjin Optical Fiber Sensing Engineering Center, Institute of Optical Fiber Sensing of Tianjin University, Tianjin 300072, China; 4 College of Physics Science and Information Engineering, Hebei Advanced Thin Films Laboratory, Hebei Normal University, Shijiazhuang 050024, China; 5 Joint Laboratory for Ocean Observation and Detection, Qingdao National Laboratory for Marine Science and Technology, Qingdao 266237, China

**Keywords:** polarization, polarimetric imaging, DoFP sensor, angle of polarization, data visualization, data analysis

## Abstract

A demand for division of focal plane (DoFP) polarization image sensors grows rapidly as nanofabrication technologies become mature. The DoFP sensor can output real time data of polarization information. In this paper, a novel visualization method for angle of polarization (AoP) is proposed for DoFP polarization image sensors. The data characteristics of AoP are analyzed, and strategies for a visualization method are proposed which conforms to the characteristics of AoP data. According to these strategies, we propose a visualization method for AoP data based on three dimensional HSI color space. This method uses intensity and saturation to characterize the magnitude of the angle between the polarization direction and the horizontal direction wherein the hue indicates the deflection direction. It is shown by the numerical simulation that the noise in the AoP image can be suppressed by our visualization method. In addition, the real-world experiment results are consistent with the numerical simulation and verify that the AoP image obtained by our method can suppress the influence of characterization noise, and the image is simple and intuitive, which is advantageous to human vision. The proposed method can be directly used for the commercialized DoFP polarization image sensor to display real-time AoP data.

## 1. Introduction

As a fundamental property of light, the polarization information can be used to obtain characteristics of an object that cannot be detected by the light intensity or wavelength information. Therefore, polarimetric imaging is widely used in the fields of underwater detection [1,2,3], biology [4,5,6], industrial inspection [7,8], remote sensing [9,10], contrast enhancement [11], etc. The polarimetric imaging system can effectively measure the Stokes parameters of the reflective light [12] or the transmitted light [13] of an object, thus achieving quantitative calculation for the polarization features of the object and, in turn, achieving optimization of contrast of the image and target identification [14]. The technique of polarimetric imaging is less susceptible to disturbance by surrounding factors and can detect the feature of the object under a complicated condition. Specifically, the division-of-focal-plane (DoFP) polarization sensor significantly decreases the system complexity of the polarimetric imaging system, improves the efficiency of polarization testing, and reduces the cost of the apparatus. Therefore, this technology becomes popular and has caused a lot of research in recent years [15,16,17,18,19,20,21].

The DoFP polarization image sensor can provide the polarization information of the scene in real time. The DoFP camera pre-stores the polarization parameters and the corresponding RGB values, and thus it is possible to convert the polarization data into image pixel values rapidly. The camera can detect and output the polarization parameters including the Stokes vector, the angle of polarization (AoP), the degree of linear polarization (DoLP), etc. Since the polarization information cannot be identified by human vision, it is necessary research on visualization of polarization data. In recent years, there are continues research results for visualization of polarization information [22,23]. The visualization method for DoLP is simple and effective. For some situations, such as mechanical processing monitoring, atmospheric remote sensing, underwater detection, etc., the objects in the situation are not significantly different in DoLP, and the AoP images herein still have a relatively high contrast and thus have a good application prospect [24,25]. However, the current visualization methods for AoP data cannot represent the AoP intuitively and effectively for human vision, and an inappropriate visualization method may introduce some image noise. Therefore, it is important to find a good visualization method for AoP data, especially with the commercial application of DoFP polarimetric sensors. The visualized AoP data can assist human in scientific research and production.

In the present paper, considering the AoP data as the research target, a novel visualization method is proposed which conforms to the AoP data structure and to human vision, and can be used to display the AoP data in image. In Section 2, the theory of polarimetric imaging and the previous visualization methods are introduced. In Section 3, the strategies which a good visualization method should meet are analyzed, and base on which a new visualization method for AoP is proposed. The method is simulated numerically. In Section 4, the applicability of our visualization method for AoP is verified by experiments. In Section 5, a conclusion is drawn.

## 2. Theory of Stokes Polarimetric Imaging

Generally, the Jones Matrix or the Stokes vector is used to describe the polarization state of light. The Stokes vector can describe both the completely polarized light and the partially polarized light, and the Stokes vector can be obtained directly by the light intensity as measured and thus is convenient to use. Therefore, the Stokes vector is the main manner for polarimetric imaging in the application field, and we describe the theory of our method by Stokes vector in the following sections.

### 2.1. Polarization Parameters and AoP Visualization Mapping Function

The mainstream DoFP sensors can only measure the linear polarization state which is dominant in nature. Therefore, the 3D Stokes vector $\left(S_{0},S_{1},S_{2}\right)$ is considered herein. The light intensities obtained by the DoFP sensor in different azimuth angles are designated as $I_{0}$, $I_{45}$, $I_{90}$ and $I_{135}$. The Stokes vector can be obtained by these light intensities:

(1) $$\begin{array}{l} S_{0}=\frac{1}{2}(I_{0}+I_{45}+I_{90}+I_{135})\\ S_{1}=I_{0}-I_{90}\\ S_{2}=I_{45}-I_{135} \end{array},$$

The AoP can be denoted by *ψ*, which can be calculated by the above Stokes vector:

(2) $$\psi =\frac{1}{2}\tan^{-1}\left(\frac{S_{2}}{S_{1}}\right),$$

The AoP has a value range of [a,b), generally $\left[0^{\circ },180^{\circ }\right)$ or $\left[-90^{\circ },90^{\circ }\right)$. A visualization method for converting the AoP data into an image corresponds to a function relation of *ψ* (as the independent variable) and the image pixel value **I** (as the dependent variable), which can be expressed as:

(3) I=f(ψ),

The previous AoP visualization method is generally divided into two categories: the grey image method and the color image method. The grey image method can be expressed as:

(4) $$I_{gray}=f_{gray}(\psi ),$$

The color image method can be expressed as:

(5) $$\begin{array}{l} R=f_{R}(\psi )\\ G=f_{G}(\psi )\\ B=f_{B}(\psi ) \end{array}$$

### 2.2. Polarization Parameters and AoP Visualization Mapping Function

**Figure 1:**
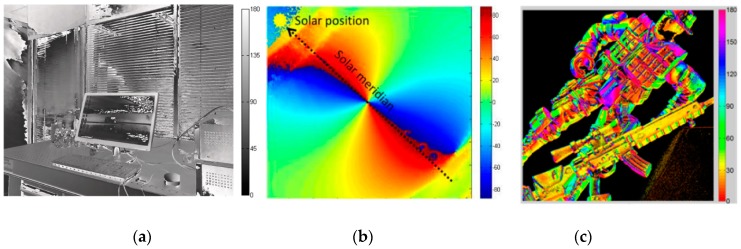
The results of previous AoP data visualization method: (**a**) Method 1 [21]; (**b**) Method 2 [26]; (**c**) Method 3 [27].

In the previous research and applications to polarimetric imaging, there are mainly three AoP data visualization methods, as shown in Figure 1. In the first method, the grey level value is used to characterize the AoP, with the state from dark to bright in the image indicating a range from 0° to 180° [21]. In the second method, various hues from blue to red are used to indicate the AoP, assuming that the saturation is 1 and the intensity is 1 [26]. In the third method, also hues, starting from a red hue and ending with another red hue, are used to indicate the AoP [27]. The first method is represented as a grey image while the other two methods are represented as a color image.

In the first visualization method, the advantage is that there is a simple linear relation between the grey level value and the AoP, and the image is intuitive and easy to understand. However, since AoP is circular data while grayscale is linear. Hence, the linear conversion of grayscale to AoP will create inconsistencies and inaccuracies in the presented data. In particular, the endpoints of the range of AoP (0°and 180°) actually indicate the same polarization direction, while the corresponding grey level values are different, that is, $f(0^{\circ })=0$ and $f(180^{\circ })=1$. This visualization method leads to a “jump” as the AoP around the endpoints and thus introduces a large noise into a certain region of the AoP image, as shown in Figure 1a. We name this kind of noise as characterization noise.

In the second visualization method, the AoP image is intuitive too. The “cold color” and the “warm color” correspond to different polarization inclination directions and the hue values indicate the degrees of inclination. However, such method also has a disadvantage similar to that in the first visualization method that the hue values corresponding to the endpoints of the value range of AoP are different: $f(0^{\circ })\neq f(180^{\circ })$.

In the third visualization method, there is an important advantage that the hue values corresponding to the two endpoints of the value range of AoP are same: $f(0^{\circ })=f(180^{\circ })$. The hue varies in a cyclic way similar with the AoP value. However, the disadvantage of such method is that it is not sufficiently intuitive. For human’s vision sense, the difference in color cannot be equivalent to the difference in magnitude. As shown in the color bars of Figure 1c, the AoP values in the range of 0° to 90° correspond to four colors: red, yellow, green and cyan. It is difficult for a person without training to obtain the AoP information from these colors, especially from a complicated image.

Each of the above three visualization methods has its own advantages and disadvantages but cannot represent the AoP well.

## 3. AoP Visualization Strategies and Method

In 2018, it is proposed by Andrew, et al. that the visualization for the polarization data should be determined not only according to the data type, the situation, or the like, but also in combination with specific application [23]. There are various display schemes for representing the polarization information. For example, Wolff et al. have first proposed that the HSL is used to describe AoP, DoLP and light intensity [28]. Gagnon et al. have proposed a method using the well-known polarization ellipse to intuitively describe the states of the polarized light [29]. In this paper, we focus on the visualization method of AoP for DoFP sensors. AoP indicates the polarimetric azimuth of light. According to the physical meaning thereof, the AoP data visualization method suitable for human’s vision sense, I=f(ψ) in the range of [a,b), should meet the following strategies:

1. f(ψ) is continuous in [a,b).
2. *ψ* and **I** correspond to each other in a one-to-one manner.
3. $\underset{\psi \to a^{+}}{\lim}f(\psi )=\underset{\psi \to b^{-}}{\lim}f(\psi )$. AoP has the same physical meaning at the two endpoints of the definition domain, so the right limit of function at left endpoint should be the same with the left limit of function at right endpoint.
4. The visualization method can be used to intuitively represent the magnitude and direction of the AoP.

According to the four strategies above, we will propose a new AoP visualization method.

Firstly, it can be proved that the visualization method with *ψ* characterized by one dependent variant cannot simultaneously meet the strategies 1), 2) and 3). Such visualization method corresponds to a simple unary function mapping: I=f(ψ). According to $\underset{\psi \to a^{+}}{\lim}f(\psi )=\underset{\psi \to b^{-}}{\lim}f(\psi )$ in the strategy 3), f(ψ) being continuous in the strategy 1) and the mean value theorem for the continuous function, it can be known that any $I\in \left(I_{\min},I_{\max}\right)$ corresponds to at least two AoP values, and thus does not meet the strategy 2). Therefore, in order to simultaneously meet the strategies 1), 2) and 3), at least a 2D vector is needed to characterize the AoP. The grey level $I_{grey}$ cannot represent the AoP well, mainly because of the periodicity of the AoP.

According to the above analysis, we find a general method which can simultaneously meet the strategies 1), 2) and 3): In any color space with RGB distributed continuously, a set of points on any one closed curve can have a nature of periodicity and can be used to characterize the AoP. It should be noted that the second previous visualization method does not use a closed curve and thus does not meet the strategy 3). The third previous visualization method uses a closed circular loop on the HSI color space (*I* = 1, *S* = 1) and thus meets the strategies 1), 2) and 3). However, in such method, the characterization image has six colors in full hue, and for human’s vision sense, there is no intuitive correspondence between these colors and the AoP magnitude.

**Figure 2:**
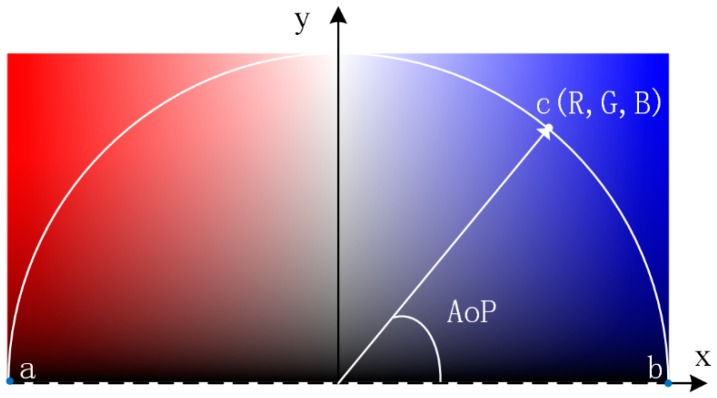
Relation diagram between AoP and **I** (*R*, *G*, *B*) according to our AoP data visualization method.

According to the above strategies, we propose a visualization method for converting AoP data into image. A color space is established as shown in Figure 2. The red component gradually decreases and the blue component gradually increases from left to right. The intensity logarithmically decreases from top to bottom, which is consistent with characteristics of the human visual system [30]. On this space, a circular arc is made with a radius of 1. The AoP is formed between a ray and the *x* axis, and the (*R*, *G*, *B*) values at the intersection point between the ray and the circular arc are the corresponding characterization values. It should be noted that on the *x* axis, the intensity *I* = 0, and thus the (*R*, *G*, *B*) value at each point between *a* and *b* is (0, 0, 0). The circular arc $\overset{⌢}{acb}$ is mapped to the RGB space to represent a closed curve. We use the set of points on such closed curve to indicate the AoP. This visualization method not only meets the strategies 1), 2) and 3), but also provides a more intuitive characterization: the intensity decreased as the polarization direction comes closer to the horizontal direction and increased as the polarization direction comes closer to the vertical direction. The different inclination directions are indicated by different colors, red and blue.

The correspondence between the characterization parameters RGB and AoP is:

(6) $$\begin{array}{l} R=\{\begin{array}{cc} \left(1-\cos\psi )\log_{10}[10\cdot (\frac{\sin\psi }{1.1}+0.1)\right] & \left[0,\frac{\pi }{2}\right)\\ \log_{10}[10\cdot (\frac{\sin\psi }{1.1}+0.1)] & \left[\frac{\pi }{2},\pi \right) \end{array}\\ G=\{\begin{array}{cc} \left(1-\cos\psi )\log_{10}[10\cdot (\frac{\sin\psi }{1.1}+0.1)\right] & \left[0,\frac{\pi }{2}\right)\\ \left(1+\cos\psi )\log_{10}[10\cdot (\frac{\sin\psi }{1.1}+0.1)\right] & \left[\frac{\pi }{2},\pi \right) \end{array}\\ B=\{\begin{array}{cc} \log_{10}[10\cdot (\frac{\sin\psi }{1.1}+0.1)] & \left[0,\frac{\pi }{2}\right)\\ \left(1+\cos\psi )\log_{10}[10\cdot (\frac{\sin\psi }{1.1}+0.1)\right] & \left[\frac{\pi }{2},\pi \right) \end{array} \end{array}$$

According to the correspondence between RGB and HSI,

(7) $$\begin{array}{l} I=(R+G+B)/3\\ H=\{\begin{array}{cc} \theta & B\leq G\\ 360-\theta & B>G \end{array}\\ S=1-\frac{3\min(R,G,B)}{R+G+B} \end{array}$$

where $\theta =\arccos\left\{\frac{\frac{1}{2}[(R-G)+(R-B)]}{{\left[{\left(R-G\right)}^{2}+(R-B)(G-B)\right]}^{\frac{1}{2}}}\right\}$, we can obtain the relation of the hue, the saturation and the intensity varying with the AoP:

(8) $$(H,S,I)=\{\begin{array}{cc} \left(0,\frac{\cos\psi }{3-2\cos\psi },(1-\frac{2}{3}\cos\psi )\log_{10}[10\cdot (\frac{\sin\psi }{1.1}+0.1)]\right) & \left[0,\frac{\pi }{2}\right)\\ \left(\frac{4}{3}\pi ,\frac{-\cos\psi }{3+2\cos\psi },(1+\frac{2}{3}\cos\psi )\log_{10}[10\cdot (\frac{\sin\psi }{1.1}+0.1)]\right) & \left[\frac{\pi }{2},\pi \right) \end{array},$$

**Figure 3:**
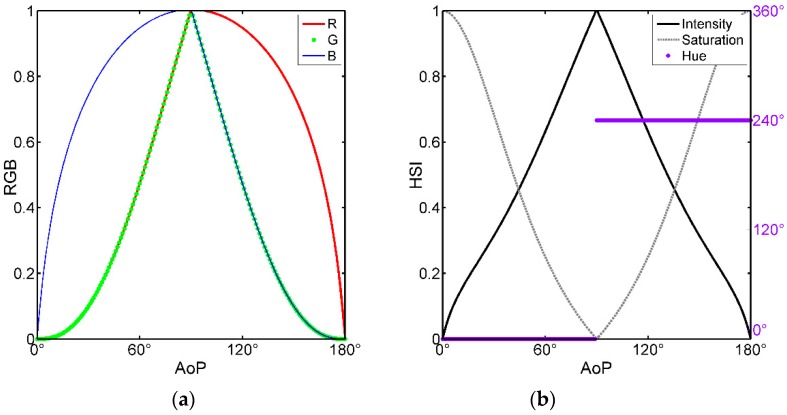
(**a**) The variation of RGB value with AoP in our method; (**b**) The variation of HSI value with AoP in our method.

The curves of the RGB and the HSI varying with the AoP are shown in Figure 3. Obviously, it can be seen from the RGB curve that the vector (R,G,B) is continuous, corresponding in one-to-one way, and equal in value at the boundaries. In the HSI curve, the hue function curve is interrupted at 90° where the saturation is 0 and the image is shown as pure white. Therefore, a sudden change in hue will not affect continuity for AoP characterization. Similarly, the intensities are 0 at the two ends of the value range of AoP, and thus the characterization values at the two endpoints are also continuous.

**Figure 4:**
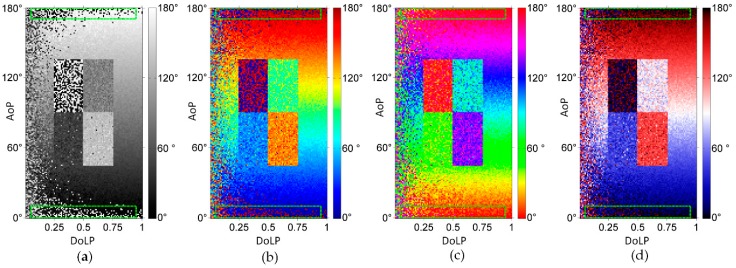
The numerical simulation results for different method. (**a**) AoP image by previous method 1; (**b**) AoP image by previous method 2; (**c**) AoP image by previous method 3; (**d**) AoP image by our method.

We use three previous visualization methods and the proposed method to perform numerical simulation, as shown in the Figure 4. The simulation region is divided into a background and four rectangular target regions. The background is a gradually changing region, with the DoLP varying from 0 to 1 in the direction from left to right and the AoP varying from 0° to 180° in the direction from bottom to top. In the middle of the scene, four objects with DoLP of 0.2 have their AoP of 0°, 30°, 60° and 90°, from top to bottom and from left to right. In the simulation, the Muller matrix of polarizer is calculated, and the intensity image measured by sensor is obtained. During the measurement of light intensity, the noise variance is 0.001, and the intensity is 1. By substituting these intensity images into the Equations (1) and (2), the AoP value is obtained and shown in Figure 4.

As can be seen from Figure 4, when the DoLP is relatively small (i.e., near the *y* axis), the noise is very large in each of the AoP images. The reason can be explained as follows: When the DoLP is relatively small, each of the Stokes components is relatively small. As can be known from the Equation (2), *ψ* is significantly affected by the noise from *S*_1_, especially when $\left|S_{1}\right|\leq \sigma$ (wherein the *σ* is the noise standard deviation of *S*_1_), by the influence of the noise, the value of *S*_1_ will change between a positive value and a negative value, and the value of *ψ* will change sharply.

It should be noted that the noise is very large in the region defined with green dotted lines in Figure 4a,b, but is much lower in the corresponding regions in Figure 4c,d. This is the characterization noise due to the fact that the visualization method does not meet the strategy 3). The reason is that near the AoP boundary, the noise causes the AoP to vary beyond the boundary, the corresponding characterization value changes from minimum to maximum or from maximum to minimum, representing a very large noise in the image. In the third method and our method, such type of noise does not occur.

## 4. Experimental Results and Discussion

**Figure 5:**
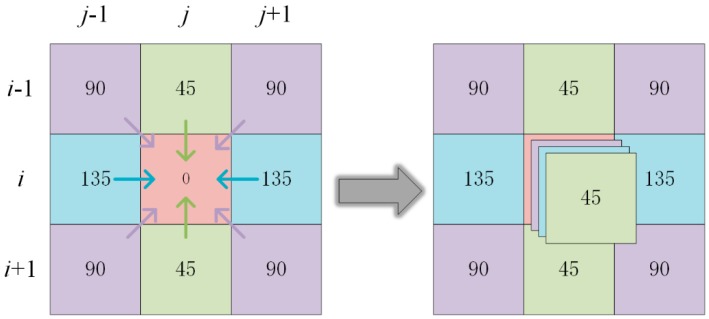
Diagram of bilinear interpolation method in DoFP polarization image sensor.

Under conditions of passive illumination, polarimetric imaging experiments are performed by a DoFP polarization image sensor (Lucid Vision labs, PHX050S-P). The DoFP polarization image sensor can measure the light intensity in only one polarization direction for any one pixel. Therefore, in order to reduce the spatial position matching error, it is necessary to use interpolation to obtain the light intensities in four polarization directions for any pixel point [31]. Different data noises are obtained by different interpolation methods, such as bilinear, bicubic, bicubic spline and gradient-based interpolation methods [27,32]. The present paper studies on the advantages and disadvantages of different visualization methods, and the conclusion can be obtained as long as the same AoP data are used. Therefore, we select the bilinear interpolation method with the simplest algorithm. Figure 5 shows a typical 3 × 3 pixel neighborhood of a DoFP polarization image sensor, and each pixel is provided thereon with polarizers in different directions. At the pixel (*i*, *j*), the light passes through the polarizer of 0° azimuth angle and is then received by the sensor, obtaining the light intensity. At this pixel point, the light intensities for the polarizer directions of 45°, 90° and 135° can be calculated by means of the Equations (9)–(11) to obtain the estimated values.

(9) $$I_{45}(i,j)=\frac{1}{2}[I_{45}(i-1,j)+I_{45}(i+1,j)],$$

(10) $$I_{90}(i,j)=\frac{1}{4}[I_{90}(i-1,j-1)+I_{90}(i-1,j+1)+I_{90}(i+1,j-1)+I_{90}(i+1,j+1)],$$

(11) $$I_{135}(i,j)=\frac{1}{2}[I_{135}(i,j-1)+I_{135}(i,j+1)].$$

**Figure 6:**
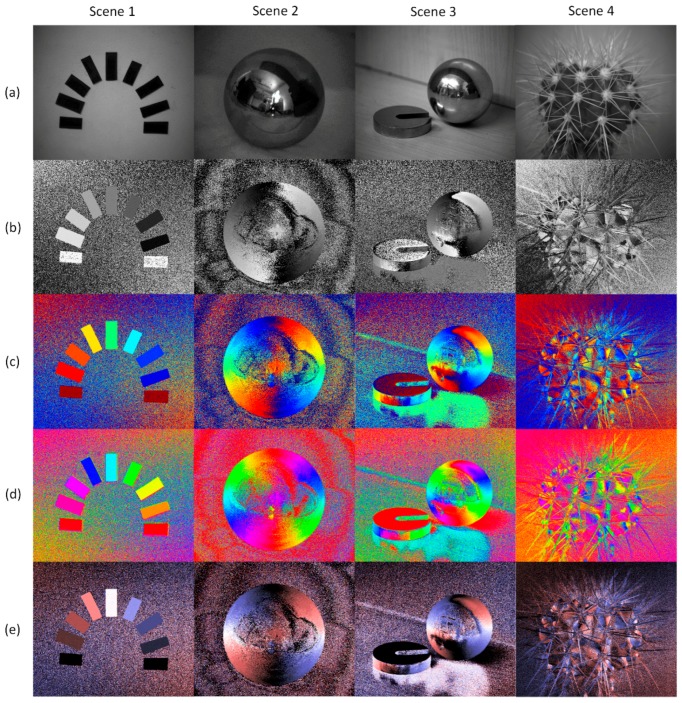
Experiment results for AoP visualization method. (**a**) Light intensity image; (**b**) AoP image by previous method 1; (**c**) AoP image by previous method 2; (**d**) AoP image by previous method 3; (**e**) AoP image by our method. The characterization noise is suppressed in (**d**) and (**e**). The AoP images are intuitively in agreement with their shape in (**c**) and (**e**), especially in the complex scene 4.

**Figure 7:**
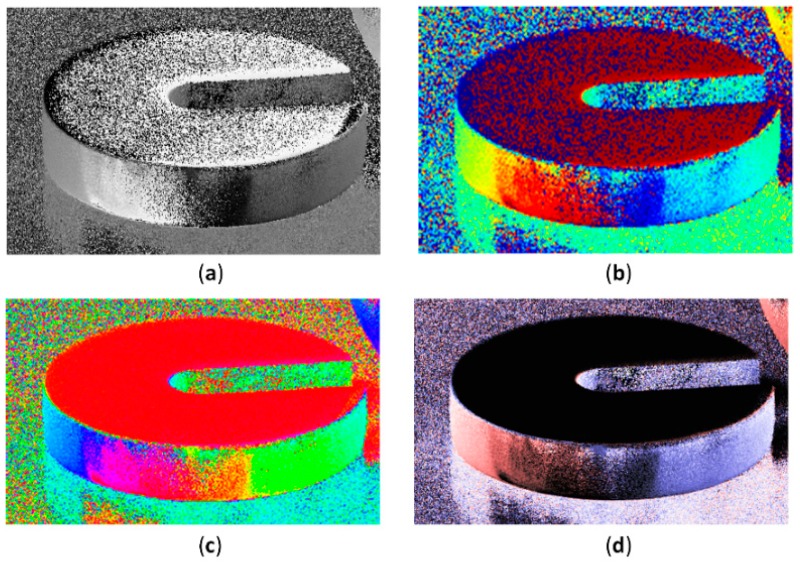
The enlarged view of Figure 6, which shows the characterization noise on the upper surface of the weight. (**a**) Previous method 1; (**b**) Previous method 2; (**c**) Previous method 3; (**d**) Our method.

The results of the experiments are shown in Figure 6, wherein four scenes are considered. In the first scene, the polarizers are placed in different directions from 0° to 180°. In the second scene, a smooth metal ball is placed on a sheet of paper which is slightly rugged. In the third scene, a metal ball and a metal weight are placed on a wooden table surface. In the fourth scene, there is a cactus with elliptic stems. By means of the visualization method mentioned in Section 3 herein, different AoP images are provided. The first line (a) shows the images for light intensity. The second, third and fourth lines (b, c and d) show the AoP images obtained by the first, second and third previous visualization methods, respectively. The fifth line shows the AoP images obtained by our method. Figure 7 shows enlarged views of the portion of the weights in the third scene.

As shown in Figure 6e, in the AoP images obtained by our visualization method, the intensity characterizes the angle between the polarization direction and the horizontal axis. The image has the minimum intensity when the polarization direction is horizontal, and has the maximum intensity when the polarization direction is vertical. Two different hues (i.e., red and blue) are used to indicate the deflection directions of the AoP, wherein blue refers to the AoP in the first quadrant and red refers to the AoP in the second quadrant. It is worth noting that the AoP of light encodes information of the three-dimensional shape of the object. Therefore, from the AoP image, the angular distribution of the target can be intuitively understood.

It can be seen from Figure 6 and Figure 7 that:

1) The AoP image can represent more information than the light intensity image. According to the theory of Fresnel reflection, the azimuth angle of metal surface determines the AoP of the reflected light [6]. As can be seen from Figure 6, different AoP values correspond to different azimuth angles of the metal surface, which has significant meaning in mechanical processing and testing, such as detecting the defects of metal surface [33].

2) There is characterization noise in the AoP image obtained by the first and second methods. The characterization noise occurs in the region where the AoP value is close to the ends of the range of AoP, such as the polarizer horizontally placed in the first scene and the upper surface of the weight in the third scene in Figure 6. In Figure 7, on the upper surface of the weight (where the polarization direction is almost horizontal), a relatively large characterization noise occurs in the first and second visualization methods, while there is no such problem in the third visualization method and our method. This is consistent with our simulation results. In the present region, the AoP is near zero, corresponding to the lowest region in the scene of Figure 4. Likely, the noise level is high for methods 1 and 2, and is low for method 3 and our method. The reason for this phenomenon lies in that the noise may cause the AoP near zero to vary between a positive value and a negative value. As the first two methods is not continuous in the characterization value at two ends of the AoP, the characterization value will vary between the maximum and the minimum, thus producing a strong characterization noise.

3) In the region of the metal ball in the second scene in Figure 6 and in the region of the side surface of the weight in Figure 7, the AoP value gradually varies from 0° to 180°. Thus, the AoP image obtained by the third method changes 6 times in color. The colors of the AoP image by the third method are over-complicated and cannot intuitively correspond to the AoP values that are related to the azimuth of the object surface. Especially in complex scenes, our method can intuitively reflect variation in AoP value, which can be seen from the images of the scene 4 in Figure 6. However, it needs to be clarified that compared with method 3, the sensitivity of AoP for our method is lower than that of method 3, as shown in Figure 7, which is because our method employs less colors to distinguish the AoP.

Our method is compared with the three previous AoP visualization methods, as shown in Table 1. Both our method and the third previous method can suppress the characterization noise. However, the third previous method uses hue to characterize AoP values, and the color variation cannot intuitively represent the variation in magnitude of AoP, with respect to human’s vision sense. In our method, there are three characterization dimensions of HSI for representing AoP data. The intensity and the saturation, which can be visually sensed in degree, are used to indicate the angle between the polarization direction and the horizontal direction; and two different colors are used to distinguish the polarization inclination directions. Therefore, the AoP can be intuitively obtained visually.

## 5. Conclusions

By studying on the AoP data structure herein, the visualization strategies for AoP are proposed. According to this, a novel AoP visualization method is proposed based on three dimensional HSI color space, which is suitable for human vision. It is demonstrated by numerical simulation and experiments that, by our method, the influence of characterization noise in the previous methods can be effectively suppressed in the AoP image and the direction of the polarization can be intuitively represented. The proposed visualization method can be directly employed for the commercialized polarization sensor, especially for DoFP polarization image sensor to display real-time AoP data.

## Figures and Tables

**Table 1:** Comparison of AoP visualization Methods.

AoP visualization method	1	2	3	Ours
Characterization noise	exist	exist	no	no
HSI characterization dimension	1	1	1	3
Intensity range	(0, 1)	1	1	(0, 1)
Saturation range	Not available	1	1	(0, 1)
Hue range	Not available	(0, 4π/3)	(0, 2π)	0, 4π/3
Number of colors in AoP image	0	4	6	2
